# Comparison of craniotomy and limited membranectomy with conventional techniques in the treatment of chronic subdural hematoma

**DOI:** 10.55730/1300-0144.5699

**Published:** 2023-07-29

**Authors:** Mehmet Emin AKYÜZ, Mehmet Hakan ŞAHİN, Mehmet Kürşat KARADAĞ

**Affiliations:** Department of Neurosurgery, Faculty of Medicine, Atatürk University, Erzurum, Turkiye

**Keywords:** Chronic subdural hematoma, membranectomy, recurrence, craniotomy

## Abstract

**Background/aim:**

Chronic subdural hematoma is the most frequently operated on intracranial hemorrhage worldwide. Although surgical results are satisfactory, recurrence remains an important problem. In this study, it was aimed to evaluate patients who underwent craniotomy with limited membranectomy (CwLM) in terms of recurrence rate and other complications.

**Materials and methods:**

Electronic records of 291 consecutive patients who were operated on at our clinic (Atatürk University Yakutiye Research Hospital) between 2015 and 2020 were retrospectively reviewed. Their radiological images at the time of admission, clinical status, any early postoperative complications, and clinical status at the 6-month follow-up were all evaluated.

**Results:**

According to the results of the study, postoperative mortality and morbidity of patients who underwent CwLM were similar to those with minimally invasive methods, and it reduced recurrence, especially in laminar and separated subtypes.

**Conclusion:**

It is our belief that CwLM will be an appropriate treatment approach in suitable patients (radiologically detected) in chronic subdural hematoma, where recurrence is an important problem.

## 1. Introduction

Chronic subdural hematoma (CSH) is the most commonly operated on intracranial hemorrhage worldwide, involving processes of inflammation and angiogenesis, particularly among the elderly population [1]. Despite advances in surgical and medical treatment, complications following surgical intervention may develop because of the advanced age of the patients and accompanying additional medical problems. Recurrence is the most significant of these complications, and it has been reported in the literature that 3%–26.5% of recurrence develops after the first intervention [2]. For the initial treatment of CSH, different surgical methods have been described, including burr hole craniostomy, craniotomy without membranectomy, and craniotomy with membranectomy (CWM) [3]. The most appropriate treatment approach is controversial among researchers.

CSH is a complex neurological entity, and its incidence increases with increasing age of the population. Moreover, the use of more anticoagulants and antiaggregants in the advanced age group increases the risk of developing CSH after minor trauma [4]. There is no subdural space, as is generally accepted [5]; the dura mater and arachnoid mater are connected to each other through a separate group of cells called dural border cells [6]. Posttraumatic blood or cerebrospinal fluid creates a space between the dura and the border cell layer [7]. The dural border cells are induced to proliferate as this subdural space is formed first, continuing to form a thin membrane and subsequently neomembranes. These neomembranes are granular tissue that is constantly proliferating. Blood vessels, which are typically missing on the dura-arachnoid surface, are a constant and are an extremely fragile feature of neomembranes. Due to the fragility of these vessels, intramembranal rebleeding continues and contributes to membrane formation [1].

Particularly, the outer membrane is adherent to the dura and is vascular in nature, with ample amounts of vEGF [8], and exudation released from the macrocapillaries is critical for CSH expansion [9]. Fenestration of the outer membrane or complete/incomplete resection may prevent postsurgical rebleeding and even facilitate efflux of the effusion in the subdural space via the dural lymphatics [10].

In terms of postoperative early and long-term clinical output, complications, and particularly recurrence, relatively minimally invasive methods like drainage from a single burr hole under the bedside or general anesthesia and gross surgical interventions, such as wide craniotomy and resection of the inner and outer membranes, have been compared with each other. Examining the literature revealed that the number of cases of craniotomy performed by membranectomy are not high, and limited studies indicate the results of this method. In this study, patients who underwent CWM were compared with those who were treated using other methods.

## 2. Materials and methods

This study was a retrospective analysis of 291 patients who underwent surgery at our clinic (Atatürk University, Yakutiye Research Hospital) between 2015 and 2020. Medical records were accessed from electronic resources registered in our hospital database. Informed consent was obtained from all of the patients and/or their legal guardian(s). This study was initiated after the approval of our faculty’s local ethics committee (Ataturk University, Faculty of Medicine, B.30.2.ATA.0.01.00/660).

The inclusion criteria were as follows: being older than 18 years of age, having unilateral CSH, having undergone surgical treatment, and having no history of acute subdural hematoma, such as acute trauma.

Computed tomography (CT) was used for the initial diagnosis and evaluation of the patients. CSH was classified as described by Nakaguchi [11] based on CT images, homogeneous architecture (Figure A), laminar architecture (Figure B), separated architecture (Figure C), and trabecular architecture (Figure D). In addition, the midline shift (mm) and initial maximum thickness of the subdural hematoma (mm) measurements were taken from the CT images. Preoperative magnetic resonance imaging (MRI) was performed on patients with inhomogeneous CT findings.

Clinical status classification was evaluated using the Markwalder grading scale (MGS) and Modified Rankin Scale scores obtained at the time of application and at the postoperative 6-month follow-up [12].

The following were determined as exclusion criteria:

- MGS grade 0
- Acute subdural hematoma
- The presence of a minimal CSH on cranial CT that was technically not drainable surgically
- Pregnancy
- Cerebrospinal fluid shunt in situ (ventriculoperitoneal shunt)
- Bilateral CSH
- Having a neurological status that had deteriorated and thus, being scheduled for emergency surgery.

Surgical evacuation was decided for a patient presenting with neurologic symptoms and a radiologically proven CSH. Asymptomatic patients without signs of brain compression and/or midline shift on radiographic films were observed conservatively. Patients without immediate deterioration were operated on, at the latest, 1 day after general surgical preparation. As part of the surgical treatment, irrigation was performed by opening the burr hole under local anesthesia, and then closed system drainage was applied, which continued for 24–72 h. This intervention was generally applied in homogeneous architecture, laminar architecture, and separated architecture hematomas. However, a small (<4 cm) craniotomy was performed if a multilayer intrahematomal structure was detected by T2-weighted MRI or the CSH was organized, calcified, and had numerous thick membranes. In these cases, a small craniotomy was performed (the thickest part of the hematoma was identified on CT and a 4 × 4-cm craniotomy was performed), the outer membrane was opened after dural opening, and the subdural space was irrigated with saline, excised to the accessible edges of the outer membrane (the surrounding outer membrane was opened at a distance of 1 cm from the craniotomy margins), and coagulated the excision margins. The inner membrane was lifted using a handle and torn 3–5 cm, the bottom was irrigated with saline again, and the inner membrane was not resected; only its integrity was broken. Although the integrity of the inner membrane was disrupted, it was attempted to not disrupt the integrity of the arachnoid mater. Limited membranectomy was generally performed in trabecular-type hematoma, but it was also performed in other types if irrigation alone was not sufficient. All of the surgeries were performed by the same surgical team under the supervision of a senior surgeon.

The closed system drainage was continued for 24–72 h postoperatively, and control brain CT was taken on the third day. However, CT was performed immediately in patients whose symptoms, such as low level of consciousness, weakness, or headache, worsened. A craniotomy was performed to evacuate the hematoma if new bleeding (acute) was observed in the early CT scan.

Control brain CT was performed at the 3- and 6-month postoperative follow-ups. According to Torihashi et al., CSH recurrence is an increase in hematoma volume within 6 months postoperatively in the ipsilateral subdural space where the operation was performed. Later, patients with radiographically increasing hematoma and neurologic deficit due to hematoma were reoperated [13].

The Clavien-Dindo Classification was used to evaluate the postoperative complications of the patients [14].

The patients were divided into 2 groups according to the surgical intervention: Group A (burr hole craniostomy and drainage) and Group B (craniotomy with limited membranectomy). The preoperative demographic, clinical characteristics, and postoperative clinical conditions and complications of the groups were compared.

All of the patients who took anticoagulants or antiplatelets were instructed to stop taking them for 10 days preoperatively and not use them until 2 weeks postoperatively if they did not experience a neurological emergency. Low-molecular-weight heparin was used during this period.

Statistical analysis was performed using the IBM SPSS Statistics for Windows 20.0 (IBM Corp., Armonk, NY, USA). Categorical variables were expressed as numbers and percentages, and the groups were compared using the χ2 and Fisher exact test. The distribution of the variables was evaluated for normality using the Shapiro-Wilk W test. Normally distributed data comprising continuous variables were analyzed using the 2-sided unpaired t test, and nonnormally distributed data were compared using the Mann-Whitney U test. P < 0.05 was considered statistically significant.

## 3. Results

Herein, 291 patients with CSH were examined, of whom 216 were men and 75 were women, whose mean age and age range were 70.4 ± 11.3 and 38–90 years, respectively. Demographic and clinical characteristics, and laboratory tests of the patients are shown in Table 1. No significant difference was found between the groups.

When the preoperative brain CT scans were examined, it was seen that the homogenous type (35%) was operated most frequently in Group A (n = 249), while it was the separated type (64%) in Group B (n = 42). Recurrence was most frequently seen in the separated type in both groups, and when surgical techniques were compared, the recurrence rate was significantly lower in the craniotomy with limited membranectomy (CwLM) group. The preoperative midline shift was significantly higher in Group B (p < 0.05) (Table 2).

Postoperative complications in the first 3 months showed no significant difference between the groups except for recurrence. (Table 3). The recurrence rates were 10.8% and 11.9% in Group A and Group B, respectively, and there was no significant difference overall. However, when the hematoma was classified according to the Nakaguchi classification, the recurrence rate was lower when CwLM was performed for the laminar and separated type (p < 0.05).

The clinical status of the patients 6 months postoperatively was compared with the MGS and there was no significant difference between the groups (Table 4)[Table t3b-turkjmedsci-53-5-1330].

## 3. Discussion

In this study, 291 patients with CSH were divided into 2 groups, as those treated with CwLM and those treated with a minimally invasive method (burr hole craniostomy), and compared. While treating CSH, the ultimate goal is to provide complete evacuation of the subdural hematoma without recurrence, while reducing the mortality and morbidity associated with the natural history of CSH and surgical intervention. It was observed that CwLM had a similar mortality and morbidity rate compared with the minimally invasive approach during the follow-up period, the risk of developing complications was the same, and the recurrence rate was less in separated and trabecular type CSH.

The internal architecture of CSH varies from case to case, and it is especially important to evaluate the intrastructural features with MRI. When T2-weighted MRI is performed in patients with organized, calcified, or numerous thick membranes, reactive tissue can be easily selected because of its high vascularity [15]. It is our belief that an MRI scan will be useful in choosing the treatment method, especially in multilayer hematomas. The internal architecture appears to be an independent risk factor for CSH recurrence. When the literature was reviewed, the recurrence rates for separated type CSH were reported by Lee et al. as 38%, Qian et al. as 31%, and Lin et al. as 66% [16–18]. In the current study, the recurrence rate was 57%, in parallel with the literature.

One of the most important difficulties of CSH evacuation with burr hole craniostomy is that the passage between the layers cannot always be ensured, and fresh bleeding that cannot be reached and emptied is left behind, membrane structures are also left behind, which cause inflammatory and angiogenesis processes to continue [19]. The neuroendoscopic operative approach may be the solution here, but it is not available at every center, and the difficulty in accessing the endoscope in emergencies creates limitations in clinical practice [20]. For many years, it has been claimed in the literature that mortality and morbidity after membranectomy are higher than minimally invasive methods [21]. Aggressive membranectomy causes various problems, such as postoperative convulsion, intracranial hematoma, brain contusion due to excessive retraction of the cerebral cortex, and failure to coagulate the resected membrane edges [22, 23]. However, there is no difference in terms of mortality and morbidity when the outer membrane is resected up to the accessible edges, the resection margins are coagulated, and the inner membrane fenestration is performed. Kidangan et al. reported a recurrence rate of 6.25% in patients treated with bedside twist drill burr hole in their study, but there were no details given about which hematoma type it was applied to. In our study, the recurrence rate was similar, and all subtypes were analyzed [24]. It is necessary to consider the internal architecture of the CSH before evaluating the use of minimally invasive approaches. In the laminar type, the laminar structure along the inner membrane is assumed to be fresh blood from the hematoma membrane, and the high recurrence rate at this stage is considered to be due to increased vascularity more than in the homogeneous type [25]. In the separated type, the hematoma is divided into 2 components; a thin line mixes with the liquefied hematoma and normal head movement cannot homogenize this hematoma. At this stage, the hematoma continues to expand and there is a high tendency of bleeding due to active hyperfibrinolytic activity [25]. Veken et al. reported a recurrence rate of 14.6% in patients treated with minicraniotomy and confirmed that this rate was lower than that with burr hole craniostomy. They also reported similar early and long-term postoperative complications with the burr hole technique. The results they obtained confirm those in the current study. However, while the results obtained from treatment of their patients were presented in their study, the present study also presented an evaluation according to the internal architecture of the CSH, which has not previously been studied in the literature [26].

There were several limitations to this study. First, it was conducted with a relatively small patient group, in a single center, and retrospectively. Randomized control studies and/or prospective studies in larger series would be more appropriate. Although the patients were followed-up by a senior surgeon and a senior surgical team at our clinic, the fact that different surgeons continued the treatment at different times created diversity and perhaps affected the results. Pharmacotherapy (oral atorvastatin calcium or dexamethasone treatment) and embolization of the meningea media artery have been described for the treatment of CSH or its recurrence postoperatively, but these methods were not used in the patient group in this study. We believe that it may be useful to investigate the effects of these nonsurgical methods in future studies. Maximum thickness measurement was a limitation for a more precise volume analysis, more precise volume measurements should be used.

CSH is an important form of hemorrhagic stroke and not only bleeding, but also inflammatory and angiogenesis processes aggravate the condition, particularly in the elderly population. Generally, satisfactory results are obtained with an appropriate treatment approach. The recurrence rate can be significantly reduced by carefully evaluating the preoperative CT images of patients with CSH, determining the separated and laminar types according to the Nakaguchi classification, and performing craniotomy and limited membranectomy as mentioned above in this group of patients.

## Figures and Tables

**Figure:**
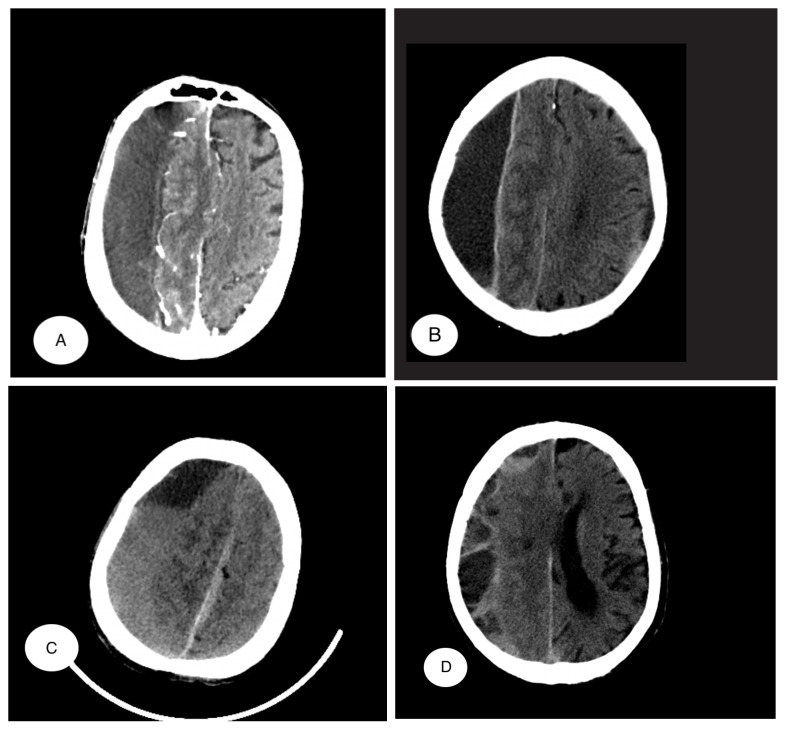
CT characteristics of CSH. (A) homogeneous architecture, (B) laminar architecture, (C) separated architecture, and (D) trabecular architecture.

**Table 1:** Comparison of the preoperative demographic, clinical, and laboratory tests of the patients. Group A: burr hole craniostomy and drainage and Group B: craniotomy with limited membranectomy.

Factors	Group A (n = 249)	Group B (n = 42)	p-value
Age (years)	70.4 ± 10.8	70.6 ± 12.7	0.304
Sex			0.313
Male/female	130/119 (52%/48%)	22/20 (52%/48 %)	
Diabetes mellitus			0.491
No/yes	165/84 (66%/34%)	24/18 (57%/43%)	
Hypertension			0.243
No/yes	114/135 (46%/54%)	23/19 (55%/45%)	
History of trauma			0.408
No/yes	120/129 (48%/52%)	20/22 (48%/52%)	
Hemoglobin (g/dL)	13.9 ± 1.8	13.9 ± 2.07	0.935
WBC (**×**10^3^/μL)	8.3 ± 2.5	8.4 ± 2.8	0.979
Platelet (**×**10^3^/μL)	256.50 ± 82.88	222.82 ± 78.75	0.069
INR	1.29 ± 0.91	1.15 ± 0.56	0.449
Markwalder grading scale			0.410
Grade 0	0 (0%)	0 (0%)	
Grade 1	195 (78.3%)	29 (69.4%)	
Grade 2	37 (14.8%)	8 (19.0%)	
Grade 3	17 (6.8%)	5 (11.9%)	
Grade 4	0 (0%)	0 (0%)	

	Group A (n = 249)	Group B (n = 42)	Recurrence rates (n = group A: 27/B: 5)	p-value
Membrane layer^*^				0.021
Homogeneous	88 (35%)	0	1/0 (2%, 0%)	
Laminar	80 (32%)	5 (12%)	13/1^*^ (25%, 10%)	
Separated	21 (8%)	27 (64%)	11/4 ^*^ (57%, 15%)	0.012
Trabecular	60 (25%)	10 (24%)	2/0 (3%, 0%)	0.014
Midline shift^*^ (mm)	4.39 ± 3.5	6.33 ± 4.38		0.025
Maximum thickness of the hematoma (mm)	19.82 ± 6.57	22.17 ± 9.31		0.163

**Table 2:** Evaluation of the internal architecture of CSHs according to the preoperative CT images.

CDG grade	Definition	Surgical complications Group A		Medical complications Group A/B	
Grade 1	Any deviation from the normal postoperative course, which can be treated without pharmaceutical, surgical, endoscopic, or radiological intervention. Excluded from these are antiemetics, antipyretics, analgesics, diuretics, electrolytes, and physiotherapy	13 (5.2%)	13 recurrences^±^	3 (1.2%)	1 decubitus, 1 urinary retention, 1 hypotension
Grade 2	Requiring treatment with pharmaceutics other than the ones allowed in grade 1	13 (5.2%)	12 epileptic seizures (first-time occurrence), 1 wound infection	24 (9.6%)	1 acute peripheral artery occlusion, 2 cardiac decompensations, 4 deliria, 1 gout relapse, 2 infection NOS, 1 thrombosis, 11 urinary tract infections, 1 gastric bleeding
Grade 3	Requiring surgical, endoscopic, or radiological treatment				
Grade 3a	Intervention not under general anesthesia	4 (1.6%)	2 wound infection, 2 wound healing disorders	4 (1.6%)	2 ascites, 2 thrombosis
Grade 3b	Intervention under general anesthesia	37 (14.8%)	5 wound infections, 7 hematoma infection, 18 recurrences, 7 wound healing disorders		
Grade 4	Life-threatening complications requiring intensive care unit (ICU) management				
Grade 4a	Single-organ dysfunction				
Grade 4b	Multi-organ dysfunction				
Grade 5	Death of the patient*			5 (2.0%)	1 liver failure, 1 pneumonia, 2 sepses, 1unknown

* p < 0.05 indicates statistical significance.

**Table 3A:** Evaluation of the complications seen in the first 3 months postoperatively with the Clavien-Dindo grading (CDG) system in Group A.

CDG grade	Definition	Surgical complications Group B		Medical complications Group B	
Grade 1	Any deviation from the normal postoperative course, which can be treated without pharmaceutical, surgical, endoscopic, or radiological interventions. Excluded from these are antiemetics, antipyretics, analgesics, diuretics, electrolytes, and physiotherapy	3 (7.1%)	1 optic hallucination (DD seizure), 1 shunt malfunction, 1 wound rebleeding	1 (2.3%)	1 delirium
Grade 2	Requiring treatment with pharmaceutics other than the ones allowed in grade 1	2 (4.7%)	2 wound infection	4 (9.5%)	1 acute peripheral artery occlusion, 2 cardiac decompensations, 1 gastric bleeding
Grade 3	Requiring surgical, endoscopic, or radiological treatment				
Grade 3a	Intervention not under general anesthesia	1 (2.3%)	1 wound infection	1 (2.3%)	1 urinary retention
Grade 3b	Intervention under general anesthesia	5 (11.9%)	3 wound infections, 2 hematoma infection		
Grade 4	Life-threatening complications requiring intensive care unit (ICU) management				
Grade 4a	Single-organ dysfunction				
Grade 4b	Multi-organ dysfunction				
Grade 5	Death of the patient*			1 (2.3%)	1 liver failure

* Deaths occurring within the first 3 months and associated with surgery were included, no statistically significant differences were observed.

± Subdural effusion or fluid accumulation that did not extend more than 1 cm in width and did not cause neurologic symptoms.

**Table 3B t3b-turkjmedsci-53-5-1330:** Evaluation of the complications seen in the first 3 months postoperatively with the CDG system in Group B.

Factors	Group A (n = 249)	Group B (n = 42)	p-value
Markwalder grading scale			
Grade 0	195 (78.3%)	33 (78.5%)	0.32
Grade 1	7 (2.8%)	1 (2.3%)	0.43
Grade 2	30 (12.1%)	5 (11.9%)	0.23
Grade 3	12 (4.8%)	2 (4.7%)	0.65
Grade 4	5 (2.0%)	1 (2.3%)	0.75
Modified Rankin scale			
Grade 0	185 (74.2%)	30 (71.4%)	0.12
Grade 1	10 (4%)	3 (7%)	0.65
Grade 2	25 (10%)	3 (7%)	0.54
Grade 3	5 (2%)	2 (4%)	0.31
Grade 4	8 (3.2%)	1 (2.3%)	0.43
Grade 5	4 (1.6%)	1 (2.3%)	0.56
Grade 6	5 (2.0%)	1 (2.3%)	0.75

* Deaths occurring within the first 3 months and associated with surgery were included, no statistically significant differences were observed.

± Subdural effusion or fluid accumulation that did not extend more than 1 cm in width and did not cause neurologic symptoms.

## References

[1] Sahyouni R, Mahboubi H, Tran P, Roufail JS, Chen JW (2017). Membranectomy in chronic subdural hematoma: meta-analysis. World Neurosurgery.

[2] Yamamoto H, Hirashima Y, Hamada H, Hayashi N, Origasa H (2003). Independent predictors of recurrence of chronic subdural hematoma: results of multivariate analysis performed using a logistic regression model. Journal of Neurosurgery.

[3] Ducruet AF, Grobelny BT, Zacharia BE, Hickman ZL, DeRosa PL (2012). The surgical management of chronic subdural hematoma. Neurosurgical Review.

[4] Ashry A, Al-Shami H, Gamal M, Salah AM (2022). Local anesthesia versus general anesthesia for evacuation of chronic subdural hematoma in elderly patients above 70 years old. Surgical Neurology International.

[5] Haines DE, Harkey HL, Al-Mefty O (1993). The “subdural” space: a new look at an outdated concept. Neurosurgery.

[6] Wittschieber D, Karger B, Pfeiffer H, Hahnemann M (2019). Understanding subdural collections in pediatric abusive head trauma. American Journal of Neuroradiology.

[7] Zhou Z, Li X, Kleiven S (2019). Biomechanics of acute subdural hematoma in the elderly: A fluid-structure interaction study. Journal of Neurotrauma.

[8] Hohenstein A, Erber R, Schilling L, Weigel R (2005). Increased mRNA expression of VEGF within the hematoma and imbalance of angiopoietin-1 and-2 mRNA within the neomembranes of chronic subdural hematoma. Journal of Neurotrauma.

[9] Aspelund A, Antila S, Proulx ST, Karlsen TV, Karaman S (2015). A dural lymphatic vascular system that drains brain interstitial fluid and macromolecules. Journal of Experimental Medicine.

[10] Bucchieri F, Farina F, Zummo G, Cappello F (2015). Lymphatic vessels of the dura mater: a new discovery?. Journal of Anatomy.

[11] Nakaguchi H, Teraoka A, Suzuki Y, Adachi S (2003). Relationship between classification of CSDH according to the Internal architecture and hematoma contents. Neurological Surgery.

[12] Miah IP, Holl DC, Peul WC, Walchenbach R, Kruyt N (2018). Dexamethasone therapy versus surgery for chronic subdural haematoma (DECSA trial): Study protocol for a randomised controlled trial. Trials.

[13] Torihashi K, Sadamasa N, Yoshida K, Narumi O, Chin M (2008). Independent predictors for recurrence of chronic subdural hematoma: A review of 343 consecutive surgical cases. Neurosurgery.

[14] Clavien PA, Barkun J, De Oliveira ML, Vauthey JN, Dindo D (2009). The Clavien-Dindo classification of surgical complications: Five-year experience. Annals of Surgery.

[15] Miah IP, Tank Y, Rosendaal FR, Peul WC, Dammers R (2021). Radiological prognostic factors of chronic subdural hematoma recurrence: A systematic review and meta-analysis. Neuroradiology.

[16] Lin CC, Lu YM, Chen TH, Wang SP, Hsiao SH (2014). Quantitative assessment of post-operative recurrence of chronic subdural haematoma using mean haematoma density. Brain Injury.

[17] Qian Z, Yang D, Sun F, Sun Z (2017). Risk factors for recurrence of chronic subdural hematoma after burr hole surgery: potential protective role of dexamethasone. British Journal of Neurosurgery.

[18] Chon KH, Lee JM, Koh EJ, Choi HY (2012). Independent predictors for recurrence of chronic subdural hematoma. Acta Neurochirurgica.

[19] Hosoda K, Tamaki N, Masumura M, Matsumoto S, Maeda F (1987). Magnetic resonance images of chronic subdural hematomas. Journal of Neurosurgery.

[20] Yan K, Gao H, Wang Q, Xu X, Wu W (2016). Endoscopic surgery to chronic subdural hematoma with neovessel septation: technical notes and literature review. Neurological Research.

[21] Sambasivan M (1997). An overview of chronic subdural hematoma: experience with 2300 cases. Surgical Neurology.

[22] Kusano Y, Horiuchi T, Seguchi T, Kakizawa Y, Tanaka Y (2010). Local brain herniation after partial membranectomy for organized chronic subdural hematoma in an adult patient: case report and review of the literature. Brain Injury.

[23] Sahyouni R, Goshtasbi K, Mahmoodi A, Tran DK, Chen JW (2017). Chronic subdural hematoma: A historical and clinical perspective. World Neurosurgery.

[24] Kidangan GS, Thavara BD, Rajagopalawarrier B (2020). Bedside percutaneous twist drill craniostomy of chronic subdural hematoma—A single-center study. Journal of Neurosciences in Rural Practice.

[25] Nakaguchi H, Tanishima T, Yoshimasu N (2001). Factors in the natural history of chronic subdural hematomas that influence their postoperative recurrence. Journal of Neurosurgery.

[26] Mahmood SD, Waqas M, Baig MZ, Darbar A (2017). Mini-craniotomy under local anesthesia for chronic subdural hematoma: an effective choice for elderly patients and for patients in a resource-strained environment. World Neurosurgery.

